# Medication Adherence and Its Determinants Among Older Adults With Chronic Illnesses in Rural and Urban Gujarat, India: A Community-Based Cross-Sectional Study

**DOI:** 10.7759/cureus.111631

**Published:** 2026-06-27

**Authors:** Nirmal Jyoti Jyotsana, Niraj Pandit, Ijaj S Aevara, Sanjana Jadhav, Yagna Pandit

**Affiliations:** 1 Community Medicine, Smt. B.K. Shah Medical Institute and Research Centre, Sumandeep Vidyapeeth (Deemed to be University), Vadodara, IND; 2 Exercise Physiology and Kinesiology, Midwestern State University, Wichita Falls, USA

**Keywords:** chronic illness, geriatric population, india, medication adherence, older adults, rural-urban comparison

## Abstract

Background

Medication adherence is essential for effective management of chronic illnesses among older adults. However, non-adherence remains a major challenge in low- and middle-income countries (LMICs), where ageing populations frequently experience multimorbidity and complex treatment regimens. This study assessed the levels of medication adherence and identified factors associated with adherence among geriatric individuals in rural and urban areas of Gujarat, India.

Methods

A community-based cross-sectional study was conducted from February 2023 to June 2024 among 380 individuals aged ≥60 years receiving long-term medication. Data were collected using a pre-tested questionnaire, and medication adherence was assessed using the 8-item Morisky Medication Adherence Scale (MMAS-8). Associations were examined using chi-square tests and multivariable logistic regression.

Results

Among 380 participants, 194 (51.1%) demonstrated high medication adherence, whereas 156 (41.0%) had low adherence. Medication adherence was significantly associated with gender, place of residence, marital status, occupation, socio-economic status, addiction status, dosing frequency, and routine follow-up behaviour on bivariate analysis. In multivariable logistic regression, higher socio-economic status (aOR = 1.91; 95% CI: 1.16-3.15) and once-daily dosing (aOR = 2.10; 95% CI: 1.19-3.70) were independently associated with better adherence. Forgetfulness was the most commonly reported reason for missed medication doses.

Conclusion

Medication non-adherence remains common among older adults with chronic illnesses. Higher socio-economic status and simpler dosing regimens were independently associated with better adherence. Interventions promoting regimen simplification and patient support may improve medication adherence in this population.

## Introduction

Ageing represents a gradual biological transition associated with declining physiological reserve and an increased susceptibility to chronic health conditions. Individuals aged 60 years and above are generally classified as geriatric [1]. Population ageing has emerged as a major demographic shift worldwide, largely attributed to improvements in healthcare services, reduced fertility rates, and longer life expectancy. As a result, the proportion of older adults requiring long-term medical care is steadily increasing [2].

Increasing age is strongly associated with a higher risk of developing chronic non-communicable diseases [3]. Many older adults experience multimorbidity, defined as the coexistence of two or more chronic conditions requiring long-term care. Evidence indicates that multimorbidity affects a substantial proportion of the ageing population globally. A systematic review and meta-analysis reported that more than half (51.0%) of adults aged 60 years and above experience two or more chronic conditions [4]. High prevalence rates have been reported in Europe and North America, as well as studies from the European Union and Scotland [5,6]. In India, a hospital-based study from Odisha and a primary care study from Kerala found substantial rates of multimorbidity, particularly among older adults. These findings underscore multimorbidity as a major clinical challenge, leading to increased healthcare needs and treatment complexities for older populations [7,8].

Polypharmacy, commonly defined as the concurrent use of five or more medications, has become increasingly prevalent among older adults, driven by population ageing, multimorbidity, and disease-specific prescribing. A large population-based database study from Scotland reported a substantial rise in medication exposure between 1995 and 2010. The proportion of individuals dispensed five or more medicines nearly doubled to 20.8%, while hyperpolypharmacy (10 or more medicines) tripled to 5.8%. These trends reflect the growing complexity of chronic disease management in ageing populations. Although multiple medications may be clinically necessary, polypharmacy is associated with higher risks of adverse drug reactions, drug-drug interactions, and treatment burden. Importantly, increasing regimen complexity and pill burden may negatively influence medication-taking behaviour, thereby contributing to suboptimal adherence among older adults [9].

Adherence to prescribed medications is essential for optimal management and control of chronic illnesses. However, adherence levels remain suboptimal in this population. Poor adherence has been linked with inadequate disease control, increased hospitalisations, functional decline, reduced quality of life, and higher healthcare costs. Adherence behaviour in older adults is influenced by multiple interrelated factors, including advancing age, cognitive decline, multimorbidity, polypharmacy, financial dependence, reduced social support, and barriers to healthcare access. Geriatric individuals frequently encounter additional challenges such as forgetfulness, limited understanding of treatment regimens, physical limitations, and inadequate caregiver support [10,11].

Despite the growing burden of chronic diseases and increasing medication use among older adults, community-based evidence on medication adherence from India remains limited, particularly in studies comparing rural and urban populations. Understanding the prevalence of medication adherence and its associated determinants is essential for strengthening geriatric care and informing health system planning. Therefore, the present study was undertaken to estimate the prevalence of medication adherence, identify its socio-demographic and treatment-related determinants, and compare medication adherence among older adults with chronic illnesses residing in rural and urban communities of Gujarat, India.

## Materials and methods

A community-based cross-sectional study was conducted between February 2023 and June 2024 in rural and urban areas of eastern Gujarat, India. The rural component was carried out in selected villages of Chhota Udepur district, while the urban component was conducted in the East Zone of Vadodara Municipal Corporation, covering all 10 urban health centres in the zone.

The study included geriatric individuals aged 60 years and above who were permanent residents of the selected areas and were receiving long-term medication for at least one chronic illness. Individuals who were critically ill, hospitalised at the time of data collection, cognitively unable to respond to the questionnaire, or unwilling to participate were excluded from the study.

The sample size was calculated using the formula: $n=\frac{Z^2pq}{d^2}$.

Assuming a prevalence of medication adherence of 45% reported by Shruthi et al. [12], a 95% confidence level ($Z = 1.96$ ), and an absolute precision of 5%, the required sample size was calculated as: $n=\frac{(1.96)^2\times0.45\times0.55}{(0.05)^2}=380.16\approx380$.

The sample was equally divided between rural (n = 190) and urban (n = 190) areas to facilitate comparison across settings.

In rural areas, 19 villages were selected using simple random sampling (lottery method) and 10 participants were recruited from each village. In urban areas, all 10 urban health centres in the selected zone were included, with 19 participants recruited from each centre. Households within each selected village or urban health centre area were listed. Systematic random sampling was then used to select households based on the required sample size, and one eligible participant was interviewed from each selected household. The sample was equally allocated between rural and urban areas (190 participants each). Since 19 villages were selected in the rural area and 10 urban health centres were available in the selected urban zone, participant allocation per cluster differed (10 participants per village and 19 participants per urban health centre) to achieve the required sample size. A larger number of rural clusters with fewer participants per cluster were included to improve representation across geographically dispersed rural populations, whereas urban participants were recruited from fewer, more densely populated urban health centre catchment areas.

Data were collected through face-to-face interviews using a pre-tested semi-structured questionnaire developed by the authors (Appendix 1). The tool was pilot tested among 12 participants outside the study area, and necessary modifications were made to improve clarity and content validity. The questionnaire captured information on socio-demographic characteristics, clinical profile, treatment characteristics, medication adherence, and addiction history. Participants were asked about the presence of addiction and, if present, its duration and type, including chewing tobacco, tobacco application on teeth, smoking, and alcohol consumption. For statistical analysis, addiction status was categorised as "Yes" or "No". Socio-economic status was assessed using the Modified BG Prasad classification.

Medication adherence was assessed using the licensed 8-item Morisky Medication Adherence Scale (MMAS-8) [13-15]. Based on MMAS-8 scoring, adherence was categorised as high adherence (score = 8), medium adherence (score 6 to <8), and low adherence (score <6).

The primary outcome variable was medication adherence as measured by MMAS-8. Independent variables included socio-demographic factors (age, gender, marital status, education, occupation, socio-economic status, place of residence), clinical characteristics (duration of illness, number of comorbidities, number of medications), treatment-related factors (dosage frequency), follow-up behaviour, and addiction history.

Collected data were entered into Microsoft Excel (Microsoft® Corp., Redmond, WA, USA) and subsequently analysed using jamovi statistical software (Version 2.6.44; The jamovi Project, Sydney, Australia). Categorical variables were summarised as frequencies and percentages. Associations between medication adherence and independent variables were assessed using the chi-square test. For bivariate analysis, age categories were regrouped to ensure adequate observations within each category and to satisfy the assumptions of the chi-square test. Variables showing statistical significance in the bivariate analysis were included in the multivariable logistic regression model to identify factors independently associated with medication adherence and to account for potential confounding. Occupational status was excluded from the final model because of its conceptual overlap and potential collinearity with socio-economic status. For multivariable analysis, socio-economic status categories were regrouped into two categories (lower/lower middle and middle & above) to facilitate model stability and interpretation, and medication adherence was dichotomised (high adherence vs. low/medium adherence). Results were expressed as odds ratios (ORs) with 95% confidence intervals (CIs). A p-value <0.05 was considered statistically significant.

Ethical approval was obtained from the Institutional Ethics Committee of the institute prior to commencement of the study. Written informed consent was obtained from all participants. Confidentiality and anonymity were maintained throughout the study, and participation was entirely voluntary.

## Results

The mean age of the participants was 70.5 ± 6.9 years. The majority of participants belonged to the 65-74 years age group (191, 50.3%). Females constituted 238 (62.6%) participants, and 224 (58.9%) were married. More than half of the participants had primary or secondary education (213, 56.1%), and the majority were housewives or retired individuals (289, 76.1%). Regarding socio-economic status, 166 (43.7%) participants belonged to the lower middle or lower socio-economic class, while 128 (33.7%) belonged to the upper or upper middle class. A history of addiction was reported by 91 (23.9%) participants (Table 1).

**Table 1:** Socio-demographic characteristics of study participants (N = 380)

Demographic characteristics	Rural	Urban	Total
	(n = 190)	(n = 190)	(N = 380)
Age (years)			
60-64	48 (25.3%)	38 (20.0%)	86 (22.6%)
65-74	82 (43.2%)	109 (57.4%)	191 (50.3%)
75-84	46 (24.2%)	34 (17.9%)	80 (21.1%)
≥85	14 (7.4%)	9 (4.7%)	23 (6.1%)
Mean age ± SD (years)	70.9 ± 7.4	70.1 ± 6.4	70.5 ± 6.9
Gender			
Male	66 (34.7%)	76 (40.0%)	142 (37.4%)
Female	124 (65.3%)	114 (60.0%)	238 (62.6%)
Marital status			
Married	114 (60.0%)	110 (57.9%)	224 (58.9%)
Widowed	76 (40.0%)	80 (42.1%)	156 (41.1%)
Educational qualification			
Illiterate	80 (42.1%)	37 (19.5%)	117 (30.8%)
Primary / Secondary	97 (51.1%)	116 (61.0%)	213 (56.1%)
Higher secondary / Graduate & above	13 (6.8%)	37 (19.5%)	50 (13.2%)
Occupation			
Daily wager / Farmer	49 (25.8%)	8 (4.2%)	57 (15.0%)
Service / Business	13 (6.8%)	21 (11.1%)	34 (8.9%)
Housewife / Retired	128 (67.4%)	161 (84.7%)	289 (76.1%)
Socio-economic class			
Upper / Upper middle	28 (14.7%)	100 (52.6%)	128 (33.7%)
Middle	32 (16.8%)	54 (28.4%)	86 (22.6%)
Lower middle / Lower	130 (68.4%)	36 (18.9%)	166 (43.7%)
Family Status			
Nuclear / Living alone	72 (37.9%)	62 (32.6%)	134 (35.3%)
Joint / 3rd generation	118 (62.1%)	128 (67.4%)	246 (64.7%)
Addiction			
Yes	44 (23.2%)	47 (24.7%)	91 (23.9%)
No	146 (76.8%)	143 (75.3%)	289 (76.1%)

Table 2 presents the item-wise responses to the MMAS-8 questionnaire among rural and urban participants. Forgetfulness-related behaviours were more commonly reported among rural participants, including forgetting to take medications (93, 48.9% vs 65, 34.2%) and forgetting to carry medications while travelling (80, 42.1% vs 60, 31.6%). A higher proportion of urban participants reported taking their medication on the previous day (186, 97.9% vs 162, 85.3%). Difficulty remembering to take all medications was reported less frequently among urban participants, with 122 (64.2%) reporting that they never or rarely experienced such difficulty, compared to 94 (49.5%) rural participants.

**Table 2:** Item-wise responses to MMAS-8 scale among rural and urban participants (N = 380)

MMAS-8 item	Rural (n = 190), n (%)	Urban (n = 190), n (%)
Sometimes forget to take medication	93 (48.9%)	65 (34.2%)
Missed medication in the past 2 weeks (other than forgetting)	30 (15.8%)	20 (10.5%)
Stopped medication without informing the doctor due to feeling worse	4 (2.1%)	0 (0%)
Forgot to carry medication while travelling	80 (42.1%)	60 (31.6%)
Took medication yesterday	162 (85.3%)	186 (97.9%)
Stopped medication when the condition felt under control	29 (15.3%)	7 (3.7%)
Felt hassled about sticking to the treatment plan	74 (38.9%)	59 (31.1%)
Difficulty remembering to take all medications		
Never/Rarely	94 (49.5%)	122 (64.2%)
Once in a while	61 (32.1%)	53 (27.9%)
Sometimes	20 (10.5%)	13 (6.8%)
Usually	14 (7.4%)	2 (1.1%)
All the time	1 (0.5%)	0 (0%)

**Figure 1:**
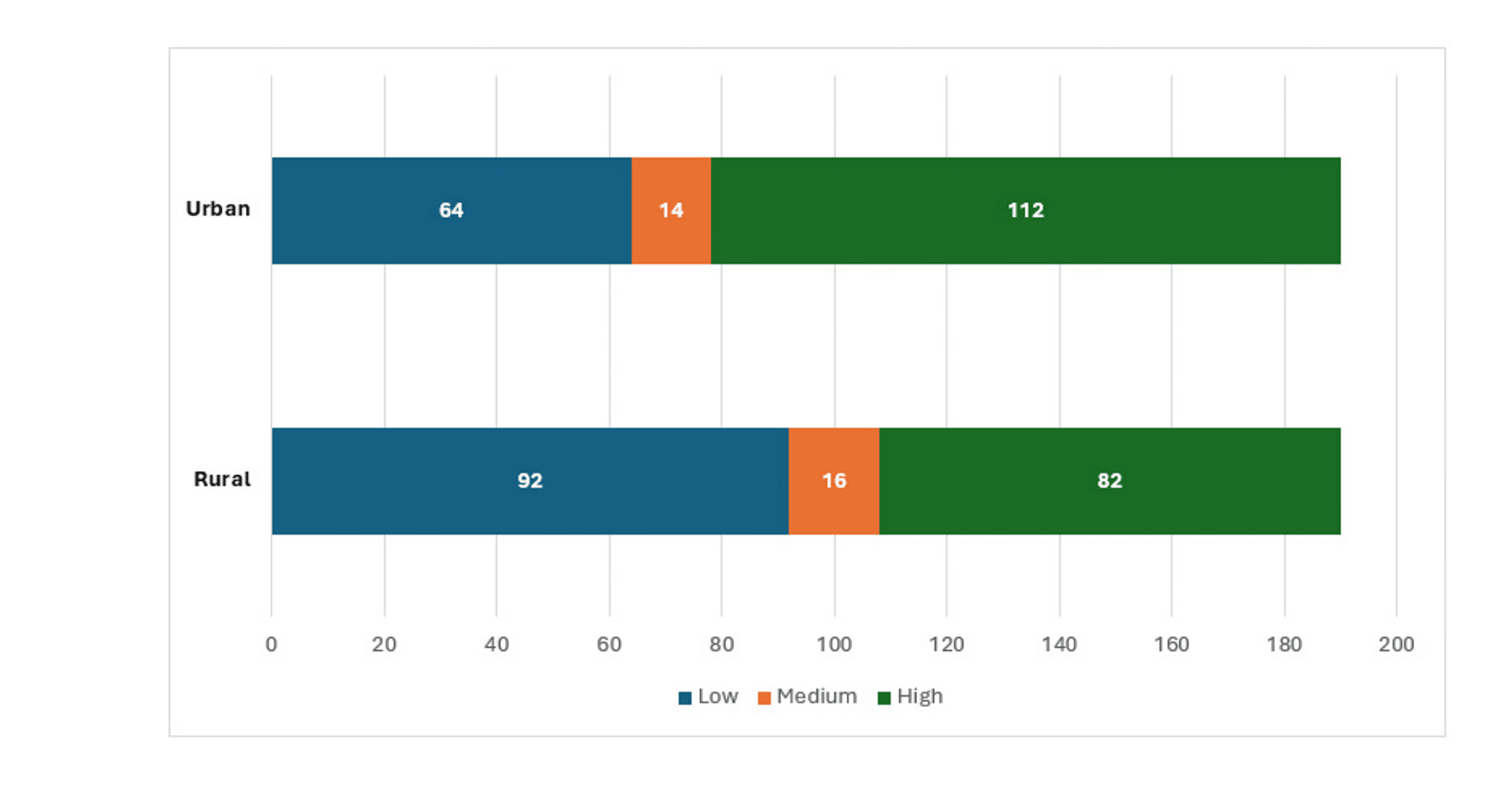
Distribution of medication adherence levels (MMAS-8) among rural and urban participants

Figure 1 illustrates the distribution of medication adherence levels based on MMAS-8 scores. High adherence was more prevalent among urban participants, whereas low adherence was more commonly observed among rural participants.

Medication adherence showed significant associations with gender (χ² = 8.25, p = 0.010), place of residence (χ² = 9.79, p = 0.007), marital status (χ² = 10.6, p = 0.004), occupational status (χ² = 12.20, p = 0.015), socio-economic status (χ² = 16.22, p = 0.002), and addiction status (χ² = 10.5, p = 0.005). Higher adherence was observed among females (135, 56.7%), urban residents (112, 58.9%), widowed participants (93, 59.6%), housewives/retired individuals (160, 55.4%), participants belonging to upper or upper-middle socio-economic classes (76, 59.4%), and those without addiction (161, 55.7%). Medication adherence also differed by place of residence, with a higher proportion of urban participants demonstrating high adherence (112, 58.9%) compared with rural participants (82, 43.2%). Conversely, low adherence was more common among rural participants (92, 48.4%) than urban participants (64, 33.7%), while the proportion of participants with medium adherence was comparable between the two groups (16, 8.4% vs. 14, 7.4%). No statistically significant association was observed between medication adherence and age (p = 0.210), educational qualification (p = 0.290), or family status (p = 0.550) (Table 3).

**Table 3:** Association between medication adherence and socio-demographic characteristics of study participants *p < 0.05 is considered statistically significant. ^#^Age categories were regrouped for bivariate analysis to ensure adequate cell frequencies for chi-square testing.

Variables	Low adherence (<6)	Medium adherence (6 to <8)	High adherence (8)	Chi-square
				p-value
Age (years)^#^				
60-70	92 (42.4%)	17 (7.8%)	108 (49.8%)	5.82; p-value = 0.210
71-80	48 (41.7%)	12 (10.4%)	55 (47.8%)	
≥81	16 (33.3%)	1 (2.1%)	31 (64.6%)	
Gender				
Male	69 (48.6%)	14 (9.9%)	59 (41.5%)	8.25; p-value = 0.010*
Female	87 (36.6%)	16 (6.7%)	135 (56.7%)	
Place of Residence				
Rural	92 (48.4%)	16 (8.4%)	82 (43.2%)	9.79; p-value = 0.007*
Urban	64 (33.7%)	14 (7.4%)	112 (58.9%)	
Marital Status				
Married	99 (44.2%)	24 (10.7%)	101 (45.1%)	10.6; p-value = 0.004*
Widowed	57 (36.5%)	6 (3.8%)	93 (59.6%)	
Educational Qualification				
Illiterate	57 (48.7%)	6 (5.1%)	54 (46.2%)	4.93; p-value = 0.290
Primary / Secondary	80 (37.6%)	19 (8.9%)	114 (53.5%)	
Higher secondary / Graduate & above	19 (38%)	5 (10%)	26 (52%)	
Occupational				
Daily Wager / Farmer	34 (59.6%)	4 (7%)	19 (33.3%)	12.20; p-value = 0.015*
Service / Business	17 (50%)	2 (5.9%)	15 (44.1%)	
Housewife / Retired	105 (36.3%)	24 (8.3%)	160 (55.4%)	
Socio-Economic Class				
Upper / Upper Middle	39 (30.5%)	13 (10.2%)	76 (59.4%)	16.22; p-value = 0.002*
Middle	33 (38.4%)	3 (3.5%)	50 (58.1%)	
Lower Middle / Lower	84 (50.6%)	14 (8.4%)	68 (41%)	
Family Status				
Nuclear / Living alone	50 (37.3%)	11 (8.2%)	73 (54.5%)	1.20; p-value = 0.547
Joint / 3rd generation	106 (43.1%)	19 (7.7%)	121 (49.2%)	
Addiction				
Yes	49 (53.8%)	9 (9.9%)	33 (36.3%)	10.5; p-value = 0.005*
No	107 (37%)	21 (7.3%)	161 (55.7%)	

Table 4 shows the association between medication adherence and disease- and treatment-related characteristics. Medication adherence was not significantly associated with duration of illness (χ² = 2.15, p = 0.708), number of comorbidities (χ² = 0.57, p = 0.749), or number of medications (χ² = 0.62, p = 0.733). However, dosing frequency demonstrated a significant association with medication adherence (χ² = 14.57, p < 0.001). Participants receiving once-daily medication regimens exhibited higher adherence, with 170 (54.3%) demonstrating high adherence, compared with 24 (35.8%) among those receiving twice- or thrice-daily regimens.

**Table 4:** Association between medication adherence and disease- and treatment-related characteristics among study participants *p < 0.05 was considered statistically significant.

Variables	Low adherence (<6)	Medium adherence (6 to <8)	High adherence (8)	Chi-square
				p-value
Duration of illness				
1 to 5 years	39 (44.3%)	8 (9.1%)	41 (46.6%)	2.15; p-value = 0.708
6 to 10 years	63 (41.4%)	9 (5.9%)	80 (52.6%)	
>10 years	54 (38.6%)	13 (9.3%)	73 (52.1%)	
Number of comorbidities				
Single	12 (48.0%)	2 (8.0%)	11 (44.0%)	0.57; p-value = 0.749
Multiple	144 (40.6%)	28 (7.9%)	183 (51.5%)	
Number of medications				
Single	69 (43.4%)	12 (7.5%)	78 (49.1%)	0.62; p-value = 0.733
Multiple	87 (39.4%)	18 (8.1%)	116 (52.5%)	
Dosing frequency				
Once daily	125 (39.9%)	18 (5.8%)	170 (54.3%)	14.57; p-value < 0.001*
Two or more daily doses	31 (46.3%)	12 (17.9%)	24 (35.8%)	

Participants who attended routine follow-up visits demonstrated significantly better medication adherence compared with those who did not attend routine follow-up visits (χ² = 6.05, p = 0.040). Among participants attending routine follow-up visits, 124 (53.2%) exhibited high medication adherence. However, the frequency of routine follow-up visits was not significantly associated with medication adherence (χ² = 0.87, p = 0.928) (Table 5).

**Table 5:** Association between medication adherence and routine follow-up behaviour among study participants *p < 0.05 was considered statistically significant.

Variables	Low adherence	Medium adherence	High adherence	Chi-square
	(<6)	(6 - <8)	(8)	p-value
Routine check-up (n = 380)				6.05; p-value = 0.040*
Yes	86 (36.9%)	23 (9.9%)	124 (53.2%)	
No	70 (47.6%)	7 (4.8%)	70 (47.6%)	
Frequency of routine check-up (n = 233)				0.87; p-value = 0.928
Every month	44 (38.3%)	11 (9.6%)	60 (52.2%)	
Every 3 months	27 (33.3%)	8 (9.9%)	46 (56.8%)	
Every 6 months	15 (40.5%)	4 (10.8%)	18 (48.6%)	

Table 6 presents the multivariable logistic regression analysis of factors associated with medication adherence. Higher socio-economic status and once-daily dosing were independently associated with better medication adherence. Other variables, including gender, place of residence, addiction, marital status, and routine follow-up, were not found to be independent predictors of adherence.

**Table 6:** Multivariable logistic regression analysis showing factors associated with medication adherence among geriatric participants (N = 380) *p < 0.05 was considered statistically significant.

Variable	Category	Crude OR (95% CI)	Adjusted OR (95% CI)	p-value
Gender	Male (Ref)	-	-	-
	Female	1.48 (0.89-2.47)	1.46 (0.88-2.44)	0.144
Place of residence	Rural (Ref)	1	1	-
	Urban	1.89 (1.26-2.84)	1.47 (0.90-2.40)	0.122
Socio-economic status	Lower / Lower middle (Ref)	1	1	-
	Middle & above	2.06 (1.37-3.12)	1.91 (1.16-3.15)	*0.011
Addiction	Yes (Ref)	1	1	-
	No	2.21 (1.36-3.60)	1.71 (0.99-2.96)	0.055
Dosing frequency	Two or more daily doses (Ref)	1	1	-
	Once daily (OD)	2.00 (1.17-3.42)	2.10 (1.19-3.70)	*0.010
Marital status	Married (Ref)	1	1	-
	Widowed	0.56 (0.37-0.84)	0.66 (0.41-1.05)	0.078
Routine follow-up	No (Ref)	1	1	-
	Yes	1.25 (0.83-1.89)	1.30 (0.84-2.02)	0.237

## Discussion

The present study assessed medication adherence and its determinants among geriatric individuals with chronic illnesses in rural and urban settings. With increasing life expectancy and the rising burden of non-communicable diseases, ensuring adequate medication adherence has become an important challenge in the management of geriatric populations. In this study, more than half of the participants (51.1%) demonstrated high medication adherence, while 41.0% had low adherence, indicating that suboptimal adherence remains common among geriatric individuals requiring long-term therapy.

Most participants belonged to the 60-69-year age group, which is comparable to findings reported in previous studies done by Vidyalakshmi et al. and Srivastava et al., where the majority of elderly participants were in early old age [16,17]. A higher proportion of females was observed in this study, similar to findings by George et al. and Hakmaosa et al., likely reflecting the greater life expectancy of women in India [18,19].

Medication adherence was significantly higher among females, urban residents, and those belonging to higher socio-economic classes. Similar gender-based differences have been reported previously, with women demonstrating better health-seeking behaviour and treatment compliance [18,19]. Better adherence among urban residents may be explained by improved healthcare accessibility, greater availability of pharmacies, higher levels of health literacy, and easier access to follow-up care compared with rural areas. Urban populations may also have better exposure to health information and stronger engagement with healthcare providers, which can positively influence medication-taking behaviour. However, although adherence was higher among urban participants on bivariate analysis, the association was no longer statistically significant after adjustment for other factors in the multivariable logistic regression model. This suggests that the observed rural-urban differences may be partly explained by underlying socio-economic and treatment-related characteristics rather than place of residence alone.

Marital status also showed a statistically significant association with medication adherence. Widowed participants demonstrated a higher proportion of high adherence (59.6%) compared with married participants (45.1%). While marital and family support have been reported to influence medication adherence among older adults, the direction of this association may vary across settings [20]. The higher adherence observed among widowed participants in the present study may reflect differences in health-seeking behaviour, self-management practices, or perceived health needs. Further research is required to better understand the factors underlying this association.

Although higher adherence was observed among participants with primary or secondary education, the association was not statistically significant. This observation suggests that formal education alone may not independently influence adherence behaviour, but likely interacts with other factors such as cognitive status, family support, and access to healthcare [12]. Higher medication adherence observed among participants belonging to upper socio-economic groups may also reflect better health literacy, greater understanding of prescribed treatment, improved access to pharmacies and healthcare services, and more proactive health-seeking behaviour. These factors may facilitate continuity of care and support better adherence to long-term medication regimens among older adults.

Using the MMAS-8 scale, 51.1% of participants showed high adherence, which is comparable to findings by Shruthi et al. and Holt et al. [12,21]. However, adherence levels were higher than those reported by Thakur et al., who observed high adherence in only 19% of participants [22]. Variations in adherence levels reported across studies may be attributed to differences in study settings, participant characteristics, healthcare accessibility, and the measurement tools used to assess adherence.

Dosage frequency emerged as a significant determinant of medication adherence. Participants receiving once-daily regimens demonstrated significantly better adherence compared to those on multiple daily doses. This finding is consistent with earlier studies highlighting that simpler treatment regimens reduce pill burden and improve compliance [12,21]. Simplified dosing schedules may improve adherence by reducing treatment complexity and minimising the likelihood of missed doses, particularly among older adults who may experience memory impairment or difficulties managing multiple medications.

Participants attending routine follow-up visits demonstrated higher medication adherence on bivariate analysis. Similar observations have been reported in previous studies emphasising the importance of continuity of care [12,23]. However, the association did not remain statistically significant after adjustment for other socio-demographic and treatment-related factors in the multivariable model. Regular follow-up visits may nevertheless reinforce medication counselling, enable early identification of treatment-related problems, and strengthen patient-provider communication, which may support adherence behaviour.

Forgetfulness was identified as the most commonly reported reason for non-adherence, reported by more than 40% of participants. This finding is consistent with earlier studies where memory impairment, lack of reminders, and inadequate family support were major contributors to missed doses [12]. This finding highlights the importance of behavioural and cognitive factors in medication adherence among older adults. Interventions aimed at improving health literacy, together with reminder-based strategies such as pill organisers, SMS reminders, mobile health applications, and caregiver-assisted reminder systems, may help support medication adherence, particularly among older adults receiving long-term therapy. However, the effectiveness of these interventions should be evaluated in future community-based studies.

The strengths of this study include its community-based design and the inclusion of both rural and urban populations, allowing comparison across different healthcare contexts. The use of a validated tool (MMAS-8) enhanced the reliability of medication adherence assessment. The study also explored a wide range of socio-demographic, clinical, and behavioural factors, providing a comprehensive understanding of medication adherence among geriatric individuals. However, the study has certain limitations. The cross-sectional design limits the causal inference between associated factors and medication adherence, and adherence was assessed using self-reported responses, which may be subject to recall and social desirability bias. Objective measures of adherence, such as pharmacy refill records or pill counts, were not available. Formal cognitive function assessment was not performed, which may have influenced participants' ability to accurately report medication-taking behaviour. Although the sampling strategy included multiple rural and urban clusters to improve representativeness, residual clustering effects cannot be completely excluded. Furthermore, the study was conducted in a single geographic region of Gujarat, which may limit the generalisability of the findings to other settings. Residual confounding due to unmeasured variables, including health literacy, disease-specific characteristics, and caregiver-related factors, cannot be excluded.

Despite these limitations, the findings provide valuable community-level evidence on medication adherence among older adults in India and emphasise the role of socio-economic and treatment-related factors in shaping medication-taking behaviour. The findings highlight potentially modifiable factors associated with medication adherence among older adults. Future studies should evaluate whether interventions such as simplified treatment regimens, improved access to medications, strengthened patient counselling, and reminder-based strategies within primary healthcare settings can improve medication adherence in this population.

## Conclusions

This community-based study highlights that poor adherence to prescribed medications continues to be an important concern among older adults living with chronic diseases. Although slightly more than half of the participants showed high adherence, a substantial proportion exhibited low adherence. Medication adherence was associated with several socio-demographic factors at the univariate level, including gender, place of residence, marital status, occupation, socio-economic status, and addiction. However, multivariable analysis identified socio-economic status and dosing frequency as the primary independent factors associated with medication adherence. Participants belonging to higher socio-economic strata and those receiving once-daily regimens had significantly greater odds of high adherence, highlighting the association of economic capacity and regimen simplicity with medication adherence.

Forgetfulness emerged as a frequently reported barrier to adherence, reflecting the behavioural and cognitive challenges commonly faced by older adults. Overall, these findings add to the limited community-level evidence on geriatric medication adherence in India and underscore the importance of considering socio-economic and regimen-related factors when evaluating adherence patterns among older adults.

## Appendices

Appendix 1

Source: Sections A, B, C, and E were developed by the authors based on the study objectives and review of relevant literature. Section D: Medication adherence was assessed using the licensed 8-item Morisky Medication Adherence Scale (MMAS-8), which is not reproduced in this appendix due to copyright restrictions. Credits: Authors.

The MMAS-8 Scale, content, name, and trademarks are protected by US copyright and trademark laws. Permission for use of the scale and its coding was obtained from MMAR, LLC. A license agreement is available from MMAR, LLC, www.moriskyscale.com.

**Table 7:** Socio-demographic profile (Section A)

No.	Particulars
A1.	Age
A2.	Gender
A3.	Place of Residence
A4.	Marital Status
A5.	Educational qualification
A6.	Occupation

**Table 8:** Family details (Section B)

No.	Particulars
B1.	Type of family
B2.	Total monthly income of family from all sources
B3.	Socio-economic status of family according to Modified B.G. Prasad Scale

**Table 9:** Addiction and history of comorbidity (Section C)

No.	Particulars
C1.	Presence of any addiction?
C1a.	If Yes, then since how long?
C1b.	Type of addiction?
C2.	Presence of any chronic illness?
C3.	Number of chronic illnesses/comorbidities
C4.	Number of medicines currently taken
C5.	Dosing frequency

Section D: Medication Adherence Assessment

Medication adherence was assessed using the licensed 8-item Morisky Medication Adherence Scale (MMAS-8). The MMAS-8 instrument is protected by copyright and is therefore not reproduced in this appendix. Participants were classified as having high adherence (score = 8), medium adherence (score 6 to <8), or low adherence (score <6) according to standard MMAS-8 scoring procedures.

The MMAS-8 Scale, content, name, and trademarks are protected by US copyright and trademark laws. Permission for use of the scale and its coding is required. A license agreement is available from MMAR, LLC., www.moriskyscale.com.

**Table 10:** Utilization and access of healthcare facility (Section E)

No.	Particulars
E1.	Do you go for routine checkup?
E2.	How frequently do you visit for routine checkup?
